# Sensitivity to thyroid hormones is associated with sleep duration in the euthyroid population with depression degree lower than moderate

**DOI:** 10.1038/s41598-024-57373-8

**Published:** 2024-03-19

**Authors:** Xian-qiu Xiao, Fu-shan Fu, Cheng Xiang, Hai-chao Yan

**Affiliations:** 1 Thyroid and Breast Surgery, The 904th Hospital of Joint Logistic Support Force of PLA (Wuxi Taihu Hospital), Wuxi, 214044 Jiangsu China; 2 Head and Neck Thyroid Surgery, The First Affiliated Hospital of Baotou Medical College of Inner Mongolia University of Science and Technology, Baotou, 014010 Neimenggu China; 3 Department of Thyroid Surgery, The Second Affiliated Hospital of Zhejiang University School of Medicine, No. 88 Jiefang Road, Shangcheng District, Hangzhou, 310009 Zhejiang China

**Keywords:** Sensitivity to thyroid hormones, Depression, Sleep, Cross-sectional analysis, Endocrine system and metabolic diseases, Thyroid diseases

## Abstract

We collected thyroid-related hormone index levels, sleep duration, and other basic characteristics of the population with depression from the NHANES 2009–2012 cycles and evaluated the association of Thyroid-Stimulating Hormone Index (TSHI) with sleep duration in the euthyroid population with depression via different analysis methods. We found that the association between TSHI and sleep duration was only found in patients with depression degree < Moderate (score: 1–14) rather than > Moderate group. Among the populations with degree < Moderate (N = 1918), only 4 indexes (parametric Thyroid Feedback Quantile Index, PTFQI, Thyrotroph Thyroxine Resistance Index, TT4RI, Thyroid-Stimulating Hormone TSH, and TSHI) reflecting the sensitivity to thyroid hormones were related to the sleep duration, with a significant non-linear relationship after adjusting for potential confounders (all P < 0.05). Trend analysis indicated that with the level increase of these 4 indexes, the sleep duration increased (all P for trend < 0.001). Further, we found that TSHI was relatively more important among the 4 indexes. Sum up, sensitivity to thyroid hormones is associated with sleep duration in the euthyroid population with depression degree lower than Moderate. Poor sensitivity referred to a longer sleep duration.

## Introduction

Sleep plays a crucial role in maintaining overall well-being and mental health^1^. Eighty percent of patients with depression suffer from sleep disturbance^2^. Inadequate sleep was associated with various negative health outcomes, including an increased risk of developing depression^3^. Roberts et al. disclosed that sleep duration < 6 h per night increased the risk for major depression^4^. Goldstone et al. found that baseline sleep disturbance was a useful predictor of depression, especially the relationship between excessive somnolence and 1-year depression was steeper for girls^5^. In addition, Sharma et al. indicated that regulation of sleep can effectively prevent postpartum depression, which may be a more effective intervention than the traditional approach of antidepressant use^6^. It follows that sleep regulation in the management of depression is extremely necessary.

The sleep duration can be influenced by some elements such as dietary nutrition^7^, sleep environments^8^, and academic achievement^9^. Recently, several studies have investigated the relationship between sleep and thyroid hormone. Thyroid hormones including serum free Triiodothyronine (FT3), free Thyroxine (FT4), and Thyroid-Stimulating Hormone (TSH) levels are generally used to reflect the thyroid function. They also influence the central nervous system including the regulation of sleep–wake cycles^10^, and play a vital role in regulating metabolism as well as energy production in the body^11^. Thyroid disorders, such as hypothyroidism and hyperthyroidism, have been found to correlate with sleep disturbances including insomnia, excessive daytime sleepiness, and altered circadian rhythms^12,13^. In addition, Wang et al. found that increased sleep duration was linked with decreased FT3 levels in the adult the US population with sleep duration less than 7 h^14^. Kessler et al. indicated the decrease in serum TSH and free T4 during human sleep restriction^15^. Those studies only focus on the relationship between thyroid-related hormones and sleep, but they did not distinct the thyroid function (normal or abnormal).

In addition, it should be noted that FT3 or TSH alone may not be sufficient to reflect the regulation of thyroid hormone homeostasis^16^. Therefore, it is necessary to explore more thyroid hormone indices to comprehensively explain thyroid status. Recent years, the sensitivity to thyroid hormones have been widely paid more attention. Sensitivity to thyroid hormones refers to the body's response to these hormones, which can be influenced by various factors, including sleep durations and disturbances^17^. The sensitivity to thyroid hormones indicators includes Thyrotroph Thyroxine Resistance Index (TT4RI), Thyroid-Stimulating Hormone Index (TSHI), Thyroid Feedback Quantile-Based Index (TFQI), parametric Thyroid Feedback Quantile Index (PTFQI) and FT3 to FT4 ratio (FT3/FT4)^18^. The first three are quantitative markers for pituitary thyrotropic function^19^. FT3/FT4 for estimating the conversion efficiency of FT4 to FT3 can be used to indirectly reflect the peripheral sensitivity of thyroid hormones. At present, few studies reported the correlation of sensitivity to thyroid hormones with sleep or depression, but there is no distinction between people with euthyroid populations and people with thyroid function disorder. Furthermore, the potential interaction between sleep duration and sensitivity to thyroid hormones has not been extensively explored in the literature on the euthyroid population with depression grade. To enrich such research, we explored the association of the sensitivity to thyroid hormones with sleep duration in euthyroid populations with depression in this study.

## Methods

### Data source and study participants

**Figure 1:**
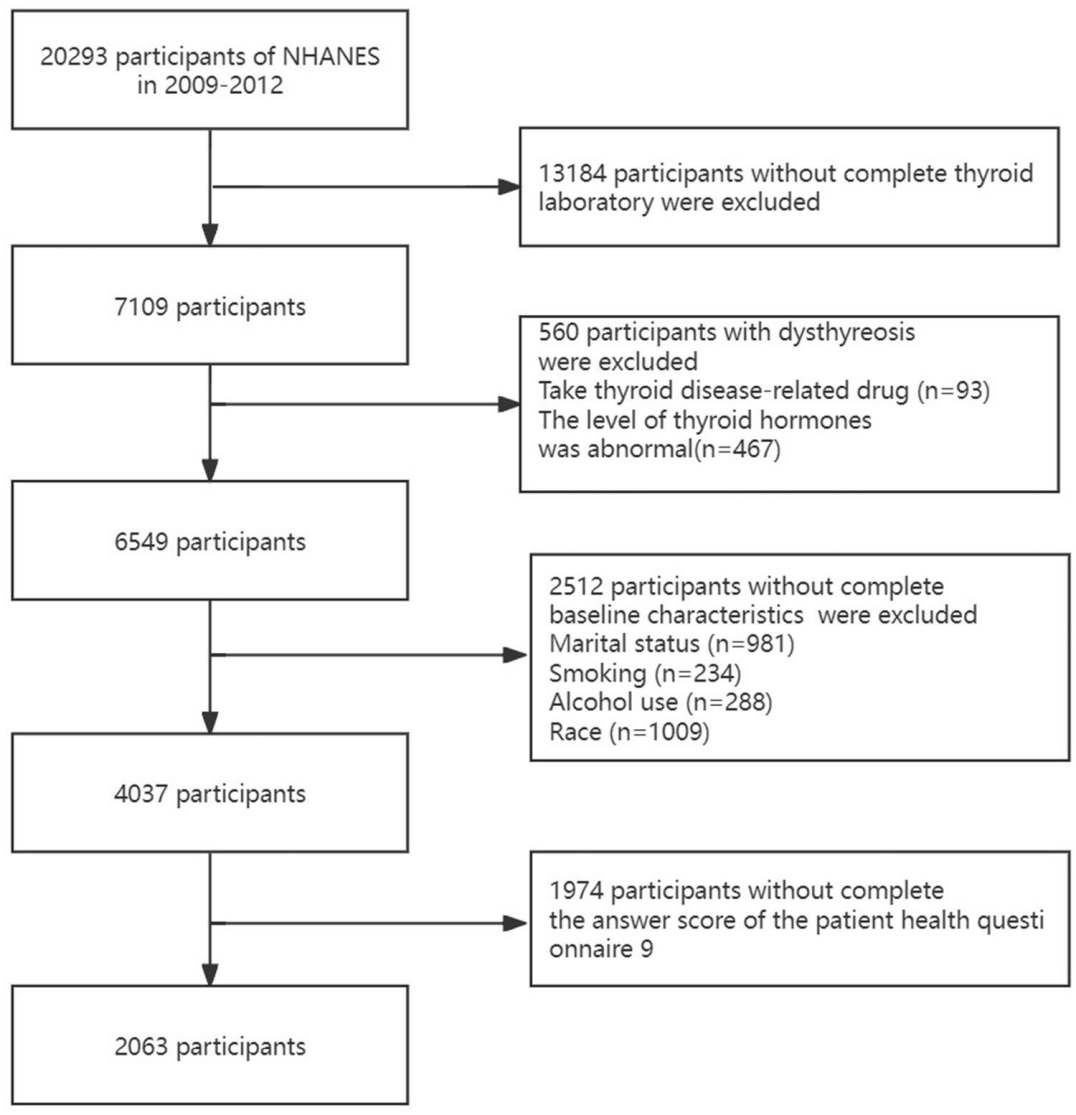
Flow charts of the inclusion and exclusion of participants.

The data used in this research were collected from the National Health and Nutrition Examination Survey (NHANES), a publicly available cross-sectional, multistage survey database, we synthesized the 2009–2010 and 2011–2012 continuous cycles data (https://www.cdc.gov/nchs/nhanes/index.htm). A total of 20,293 participants were obtained for the next screening. Then 13,184 participants whose thyroid laboratory data was incomplete, 560 participants who had dysthyreosis, 2512 participants who lacked the covariate including marital status, smoking, alcohol use, and race; and 1974 participants who did not answer the PHQ9 questionnaire were excluded. Finally, the 2063 participants were included in this study. Flow of the inclusion and exclusion of participants was showed in Fig. 1.

### Laboratory methodology and computing formula for testing thyroid function

The thyroid blood specimens was obtained form the participants who attended to in blood collection of NHANES. Thyroid blood specimens were processed, stored, and shipped to the University of Washington, Seattle, WA. Detailed specimen collection and processing instructions are discussed in the NHANES Laboratory/Medical Technologists Procedures Manual (LPM). The Access HYPERsensitive human TSH assay is a 3^rd^ generation, two-site immunoenzymatic (“sandwich”) assay. The total T3, free T3, and total T4 assay were competitive binding immunoenzymatic assays. The free T4 assay is a two-step enzyme immunoassay. The access thyroglobulin (Tg) assay is a simultaneous one-step "sandwich" assay. The thyroglobulin antibody and thyroid peroxidase antibody (TPOAb) assay is a sequential two-step immunoenzymatic "sandwich" assay. The detailed steps of the experiment are displayed on the official website (https:// wwwn. cdc. gov/ Nchs/Nhanes/ 2009- 2010/ THYROD_ F. htm) TSHI, TT4RI, TFQI, PTFQI, andT3/T4were calculated using the following formula, respectively.

$$\begin{gathered} {\text{TSHI }} = {\text{ LogTSH }}\left( {{\text{mUI}}/{\text{L}}} \right) \, + \, 0.{1345 } \times {\text{ FT4 }}\left( {{\text{pmol }}/{\text{ L}}} \right) \hfill \\ {\text{TT4RI }} = {\text{FT4}}\left( {{\text{pmol }}/{\text{ L}}} \right) \, \times {\text{ TSH }}\left( {{\text{mUI}}/{\text{L}}} \right) \hfill \\ {\text{TFQI }} = {\text{ cdf FT4 }} + {\text{ cdf TSH}} - {1} \hfill \\ {\text{PTFQI}} = \Phi \, \left( {\left( {{\text{FT4 }} - \, \mu {\text{ FT4}}} \right) \, /\sigma {\text{ FT4}}} \right) \, - \, \left( {{1 }{-} \, \Phi \, \left( {\left( {{\text{ln TSH }} - \, \mu {\text{ ln TSH}}} \right)/\sigma {\text{ ln TSH}}} \right)} \right) \hfill \\ \mu {\text{FT4 }} = { 1}0.0{75}, \, \sigma {\text{FT4 }} = { 2}.{155}, \, \mu {\text{ ln TSH }} = \, 0.{4654},{\text{ and }}\sigma {\text{ ln TSH }} = \, 0.{7744} \hfill \\ \end{gathered}$$

CDF: Cumulative distribution function.

The euthyroid individuals were screened via normality reference ranges 7.74–20.64 pmol/L for FT4 and 0.34–5.60 mIU/L for TSH^20^.

### Definitions of depression grade and sleep duration

The depression grades were defined via the answer score of the patient health questionnaire 9 (PHQ9). The PHQ9 is validated as a depressive symptom severity measure. Total score 1–4 was defined as minimal depression, 5–9 was defined as mild depression, 10–14 was defined as moderate depression, 15–19 was defined as moderately severe depression, and 20–27 was defined as severe depression^21^.

Sleep duration was obtained from the questionnaire as the follows: “Number of hours usually sleep on weekdays or workdays”. Sleep duration was categorized as < 5 h, 5–8 h, > 8 h referring to reported study^22–24^.

### Covariates

The sex, race, marital status, educational level, smoking, alcohol use, hypertension, diabetes, and stroke were collected from in-person interviews and considered as covariates in this study. The race included Mexican American, non-Hispanic White, non-Hispanic Black, other Hispanic, and other Race—Including Multi-Racial. The marital status included never marital, married /living with partner, widowed/divorced/separated. The education level included less than high school, high school or equivalent, and high school above. Smoker was defined as adults who smoked > 100 cigarettes in life and smoked some days or every day. Smokers who do not currently smoke cigarettes were considered former smokers. Never-smoker was defined as adults who smoked < 100 cigarettes in life. According to the alcohol consumption, the participants were categorized the never-alcohol, former-alcohol, and now-alcohol. Never-alcohol was defined as who drinks less than 12 in a lifetime. Former-alcohol was who met any conditions concluding (1) had above or equal 12 drinks in 1 year and did not drink last year, (2) did not drink last year but drank above or equal 12 drinks in a lifetime. The now-alcohol was defined as who drinks above or equal to 1 drink now^25,26^.

Hypertension was diagnosed according to the previous doctor's diagnosis, taking prescribed medicine to decrease blood pressure (BP), and BP of ≥ 140/90 mmHg. Stroke was diagnosed on thebasis of the answer question: “Have doctors ever told you had a stroke?”. Diabetes was diagnosed according to the question: “doctor told you had diabetes”, the use of diabetes medication or insulin, and two-hour Oral Glucose Tolerance Test (OGTT) blood glucose ≥ 11.1(mmol/L) and fasting glucose ≥ 7.0 (mmol/L).

### Statistical analyses

All the statistical analyses used the appropriate sample weights, 1/2 of two-year subsample weights according to the NHANES analysis guidelines via 4.2.2 version R software. The chi-square test was used to test for the association between categorical variables and one-way ANOVA for the association between categorical and continuous variables. The generalized additive model (GAM) and polynomial regression were used to analyze the sleep duration and depression grade. The generalized linear analysis was used to explore the relationship between the thyroid function index and sleep duration in different depression grades. Then, we found there was a relationship between sleep duration and thyroid function index in the minimal depression, mild depression, and moderate depression groups. Hence, the GAM analyses were performed to further explore the relationship between thyroid indexes and sleep duration. The generalized linear analysis was performed to evaluate the relationship between the thyroid hormone indexes with sleep duration. Model 1 was unadjusted. Model 2 was adjusted for race, education level, and BMI. Model 3 was adjusted for race, education level, BMI smoking, alcohol use, diabetes, and hypertension. Next, the trend test between the thyroid hormone indexes and sleep duration was analyzed. In addition, the four methods including Xgboost, AdaBoost, Random Forest, and light GAM were performed to evaluate the importance of thyroid hormone index.

### Ethics approval

The Ethics Committee of the 904th Hospital of Joint Logistic Support Force of PLA deemed that this research is based on open-source data, so the need for ethics approval was waived.

## Results

### Determination of study participants

**Figure 2:**
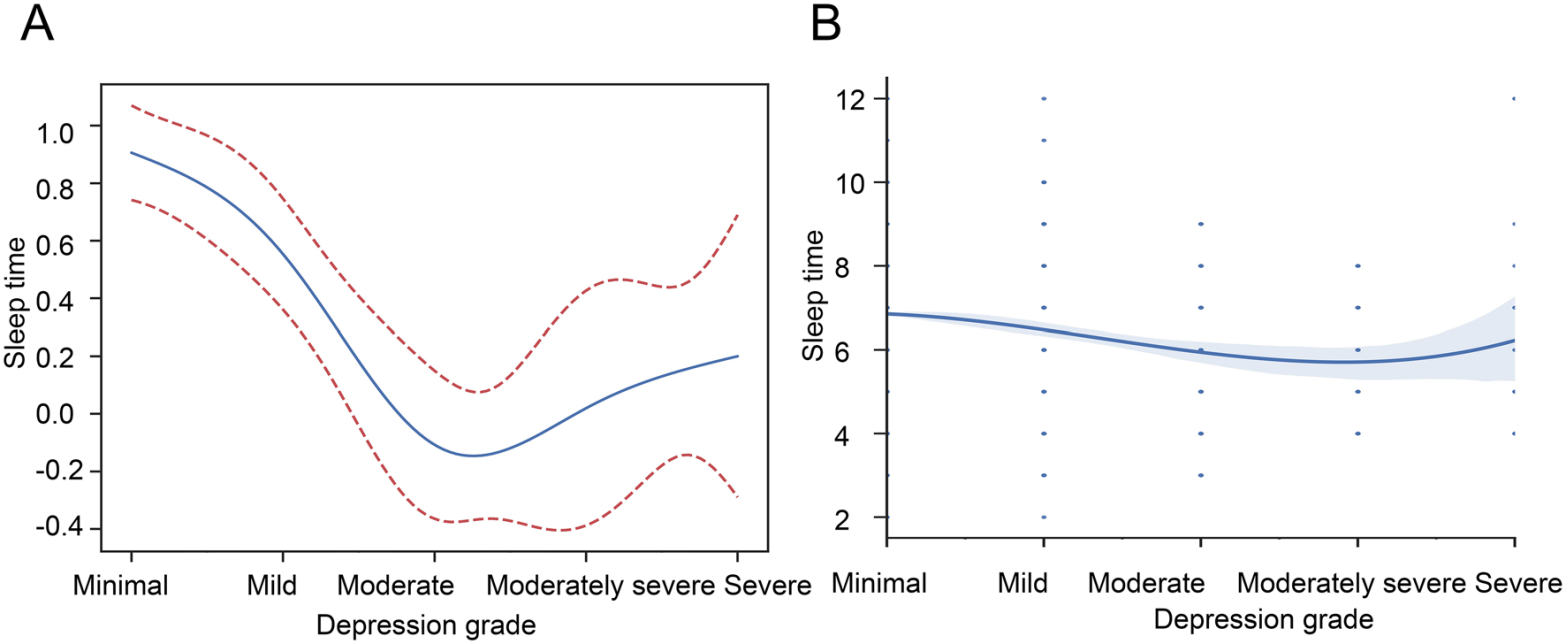
The relationship between the depression grade and sleep duration. (**A**) Generalized additive model (GAM). (**B**) Polynomial regression.

We first explored the sleep duration of populations with depression. The GAM and Polynomial regression analyses were performed to explore the correlation between sleep duration and depression grade. The results showed that there was a non-linear relationship with an inverted U-shape (Fig. 2A, P  < 0.001, β = − 0.336 [− 0.417, − 0.256]). In addition, we found a key inflection point (depression grade = Moderate). The sleep duration decreased with the depression grade increasing when the depression grade lower than Moderate. When the depression grade larger than Moderate, the sleep duration increased with the depression grade increasing. Consistent results are obtained by polynomial regression analysis (Fig. 2B).

Hence, all participants were grouped into two clusters (lower than Moderate and larger than Moderate). Next, the generalized linear model was performed to explore the relationship between the thyroid function indexes and sleep duration in two groups. The results showed that among all indicators, TSH, PTFQI, TT4RI, and TSHI correlated with the sleep duration in participants with depression grade less than Moderate (Table 1, all P < 0.001). However, no correlation was observed between sleep duration and any of indicators in participants with depression grade larger than Moderate. Therefore, the participants with depression grade less than Moderate were selected to further explore.

**Table 1:** The association between the thyroid function index and sleep duration in different depression grade group by generalized linear model.

	Less than moderate (n = 1918)			Larger than moderate (n = 145)		
	β	95% CI	P	β	95% CI	P
Total T4 (ng/dL)	− 0.011	[− 0.049, 0.028]	0.583	− 0.031	[− 0.188, 0.125]	0.694
Total T3 (nmol/L)	− 0.001	[− 0.004, 0.001]	0.280	− 0.015	[− 0.030, 0.001]	0.064
Thyroid peroxidase (IU/mL)	0.000	[− 0.001, 0.000]	0.496	− 0.002	[− 0.006, 0.002]	0.241
Thyroglobulin (ng/mL)	− 0.002	[− 0.005, 0.000]	0.078	− 0.007	[− 0.024, 0.010]	0.403
Free T4 (pmol/L)	0.001	[− 0.031, 0.033]	0.958	− 0.086	[− 0.255, 0.082]	0.316
Free T3 (pg/mL)	− 0.151	[− 0.314, 0.012]	0.069	− 0.470	[− 1.207, 0.268]	0.212
Thyroglobulin Antibodies (IU/mL)	0.000	[− 0.001, 0.001]	0.566	0.020	[− 0.027, 0.066]	0.407
TSH (mIU/L)	0.150	[0.078, 0.222]	< 0.001	0.042	[− 0.333, 0.417]	0.826
T3/T4	− 0.050	[− 0.128, 0.027]	0.201	0.057	[− 0.253, 0.366]	0.720
TFQI	0.175	[− 0.041, 0.391]	0.112	0.398	[− 0.776, 1.572]	0.506
PTFQI	0.610	[0.339, 0.880]	< 0.001	− 0.463	[− 1.533, 0.607]	0.397
TT4RI	0.014	[0.007, 0.020]	< 0.001	0.000	[− 0.031, 0.031]	0.991
TSHI	0.232	[0.120, 0.343]	< 0.001	0.021	[− 0.481, 0.524]	0.933

*PTFQI* parametric thyroid feedback quantile index, *TT4RI* thyrotroph thyroxine resistance index, *TSHI* thyroid-stimulating hormone index, *TFQI* thyroid feedback quantile-based index, *TSH* thyroid-stimulating hormone, *T4* thyroxine, *T3* triiodothyronine, *T3/T4* free T3 to free T4 ratio.

### Clinical characteristics of the participants

A total of 1918 participants with depression grade lower than Moderate were enrolled in our final analysis, and then grouped into three groups via sleep duration (< 5 h, 5–8 h, > 8 h). There were differences among the three groups in terms of PTFQI (P = 0.012), TT4RI (P = 0.015), TSHI (P = 0.023), TSH (P = 0.012), BMI (P = 0.032), race (P < 0.001), educational level (P = 0.008), smoking (P < 0.001), alcohol use (P = 0.043), hypertension (P = 0.026), and diabetes (P = 0.019) (Table 2). Only the variables with P < 0.05 were selected for further analyses.

**Table 2:** Baseline characteristics of populations with depression grades lower than moderate.

Variable	Sleep duration			F/χ^2^	p
	< 5 h (n = 110)	5–8 h (n = 1704)	> 8 (n = 104)		
Age	48.000 [35.000, 61.000]	45.000 [32.000, 60.000]	51.000 [28.000, 66.000]	3.516	0.172
PTFQI	− 0.548 [− 0.736, − 0.397]	− 0.541 [− 0.705, − 0.365]	− 0.454 [− 0.655, − 0.291]	8.82	0.012
TT4RI	15.336 [10.660, 19.200]	15.200 [10.990, 22.052]	17.978 [12.920, 25.972]	8.395	0.015
TSHI	1.786 [1.467, 2.093]	1.804 [1.457, 2.173]	1.935 [1.588, 2.325]	7.562	0.023
TSH	1.450 [0.976, 1.950]	1.470 [1.050, 2.080]	1.740 [1.170, 2.440]	8.82	0.012
BMI (kg/m^2^)	29.600 [24.420, 34.600]	27.410 [24.100, 31.780]	27.360 [23.800, 31.600]	6.882	0.032
Sex, n (%)					
Male	60 (54.545)	945 (55.458)	57 (54.808)	0.049	0.976
Female	50 (45.455)	759 (44.542)	47 (45.192)		
Race, n (%)					
Mexican American	14 (12.727)	224 (13.146)	13 (12.500)	27.553	< 0.001
Non-Hispanic White	38 (34.545)	777 (45.599)	61 (58.654)		
Non-Hispanic Black	40 (36.364)	338 (19.836)	17 (16.346)		
Other Hispanic	11 (10.000)	172 (10.094)	7 (6.731)		
Other race-including multi-racial	7 (6.364)	193 (11.326)	6 (5.769)		
Marital status, n (%)					
Never marital	23 (20.909)	358 (21.009)	25 (24.038)	6.97	0.137
Marital/living with partner	57 (51.818)	1026 (60.211)	55 (52.885)		
Widowed/divorced/separated	30 (27.273)	320 (18.779)	24 (23.077)		
Educational level, n (%)					
Less than high school	29 (26.364)	356 (20.892)	24 (23.077)	13.929	0.008
High school or equivalent	31 (28.182)	344 (20.188)	31 (29.808)		
High school above	50 (45.455)	1004 (58.920)	49 (47.115)		
Smoking, n (%)					
Never	45 (40.909)	992 (58.216)	50 (48.077)	32.064	< 0.001
Former	23 (20.909)	411 (24.120)	31 (29.808)		
Now	42 (38.182)	301 (17.664)	23 (22.115)		
Alcohol using, n (%)					
Never	15 (13.636)	194 (11.385)	16 (15.385)	9.877	0.043
Former	24 (21.818)	220 (12.911)	12 (11.538)		
Now	71 (64.545)	1290 (75.704)	76 (73.077)		
Hypertension					
No	76 (69.091)	1361 (79.871)	82 (78.846)	7.297	0.026
Yes	34 (30.909)	343 (20.129)	22 (21.154)		
Diabetes					
No	56 (50.909)	1085 (63.674)	61 (58.654)	7.954	0.019
Yes	54 (49.091)	619 (36.326)	43 (41.346)		
Stroke, n (%)					
No	106 (96.364)	1670 (98.005)	99 (95.192)	4.573	0.102
Yes	4 (3.636)	34 (1.995)	5 (4.808)		

*PTFQI* parametric thyroid feedback quantile index, *TT4RI* thyrotroph thyroxine resistance index, *TSHI* thyroid-stimulating hormone index, *TFQI* thyroid feedback quantile-based index, *TSH* thyroid-stimulating hormone, *T4* thyroxine.

### Correlation analysis between thyroid hormone indices and sleep duration

**Figure 3:**
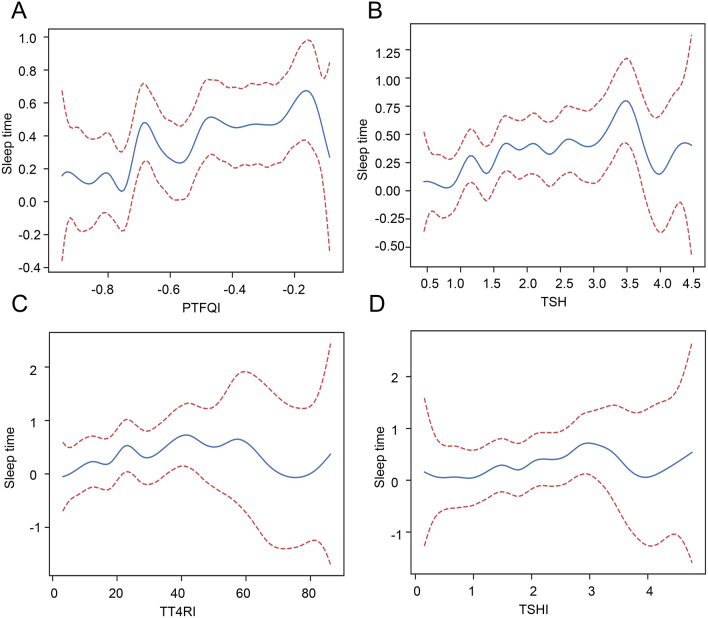
Generalized additive model analyses between the four thyroid indexes and sleep duration. (**A**) PTFQI; (**B**) TSH; (**C**) TT4RI; (**D**) TSHI.

The GAM analysis was used to preliminary explore the association of sleep duration with PTFQI, TSH, TT4RI, and TSHI. The result showed that there was a non-linear association of sleep duration with PTFQI (Fig. 3A, P < 0.001, β: 0.610, 95% CI (0.339, 0.880)), TSH (Fig. 3B, P < 0.001, β: 0.15, 95% CI (0.078, 0.222), TT4RI (Fig. 3C, P < 0.001, β: 0.014, 95% CI (0.007, 0.020)), and TSHI (Fig. 3D, P < 0.001, β: 0.232, 95% CI (0.120, 0.343)). Due to the non-linear association, the Generalized Linear Model (GLM) was performed to further explore the relationship between the thyroid hormone indexes with sleep duration. The results showed that TSHI, TT4RI, PTFQI, and TSH were significantly positively related to sleep duration (Table 3, all P < 0.001). Their relationships remained significant even after adjusting for potential confounders (all P < 0.05 both in adjusted Model 2 and Model 3).

**Table 3:** Three kinds of generalized linear analysis of thyroid hormone indexes with sleep duration (n = 1918).

Variable	Model 1	p	Model 2	p	Model 3	p
TSHI	0.232 (0.120, 0.343)	< 0.001	0.181 (0.070, 0.292)	0.001	0.163 (0.051, 0.274)	0.004
TT4RI	0.014 (0.007, 0.020)	< 0.001	0.011 (0.005, 0.018)	0.001	0.010 (0.004, 0.017)	0.001
PTFQI	0.610 (0.339, 0.880)	< 0.001	0.486 (0.216, 0.757)	< 0.001	0.442 (0.172, 0.713)	0.001
TSH	0.150 (0.078, 0.222)	< 0.001	0.122 (0.051, 0.194)	0.001	0.112 (0.04, 0.184)	0.002

Model 1: no variables adjusted.

Model 2: adjusted the race, education level, BMI.

Model 3: adjusted the race, education level, BMI smoking, alcohol use, diabetes, hypertension.

*TSHI* thyroid-stimulating hormone index, *TT4RI* thyrotroph thyroxine resistance index, *PTFQI* parametric thyroid feedback quantile index, *TSH* thyroid-stimulating hormone.

The trend test was also performed to verify the relationship degree between hormone indexes and sleep duration (Table 4). The results showed that the association between 4 indicators and sleep duration gradually increased with the increase of their levels (all crude P for trend < 0.05), and their association remained significant even after adjusting for potential confounders including race, education level, BMI smoking, alcohol use, diabetes, and hypertension (all adjusted P for trend < 0.05).

**Table 4:** The trend test between the thyroid hormone indexes and sleep duration (n = 1918).

Variable	Crude			Adjusted		
	β	95% CI	P	β	95% CI	P
TSHI						
Q1	Ref	Ref	Ref	Ref	Ref	Ref
Q2	0.045	[− 0.123, 0.212]	0.603	0.021	[− 0.146, 0.188]	0.805
Q3	0.140	[− 0.028, 0.308]	0.103	0.107	[− 0.061, 0.276]	0.212
Q4	0.292	[0.125, 0.460]	0.001	0.258	[0.089, 0.428]	0.003
P for trend	0.097	[0.044, 0.150]	< 0.001	0.086	[0.033, 0.140]	0.002
TT4RI						
Q1	Ref	Ref	Ref	Ref	Ref	Ref
Q2	0.133	[− 0.035, 0.300]	0.121	0.110	[− 0.057, 0.277]	0.197
Q3	0.168	[0.001, 0.336]	0.049	0.127	[− 0.041, 0.296]	0.138
Q4	0.348	[0.180, 0.516]	< 0.001	0.308	[0.139, 0.477]	< 0.001
P for trend	0.108	[0.055, 0.161]	< 0.001	0.094	[0.041, 0.148]	0.001
PTFQI						
Q1	Ref	Ref	Ref	Ref	Ref	Ref
Q2	0.194	[0.027, 0.361]	0.023	0.178	[0.011, 0.344]	0.037
Q3	0.328	[0.160, 0.495]	< 0.001	0.289	[0.122, 0.457]	0.001
Q4	0.376	[0.208, 0.543]	< 0.001	0.329	[0.160, 0.498]	< 0.001
P for trend	0.126	[0.073, 0.179]	< 0.001	0.110	[0.056, 0.163]	< 0.001
TSH						
Q1	Ref	Ref	Ref	Ref	Ref	Ref
Q2	0.194	[0.027, 0.361]	0.023	0.178	[0.011, 0.344]	0.037
Q3	0.328	[0.160, 0.495]	< 0.001	0.289	[0.122, 0.457]	0.001
Q4	0.376	[0.208, 0.543]	< 0.001	0.329	[0.160, 0.498]	< 0.001
P for trend	0.126	[0.073, 0.179]	< 0.001	0.110	[0.056, 0.163]	< 0.001

Crude model: no variables adjusted.

Adjusted model: adjusted the race, education level, BMI smoking, alcohol use, diabetes, hypertension.

*TSHI* thyroid-stimulating hormone index, *TT4RI* thyrotroph thyroxine resistance index, *PTFQI* parametric thyroid feedback quantile index, *TSH* thyroid-stimulating hormone.

### Verifying the importance of thyroid hormone indices affecting sleep duration

**Figure 4:**
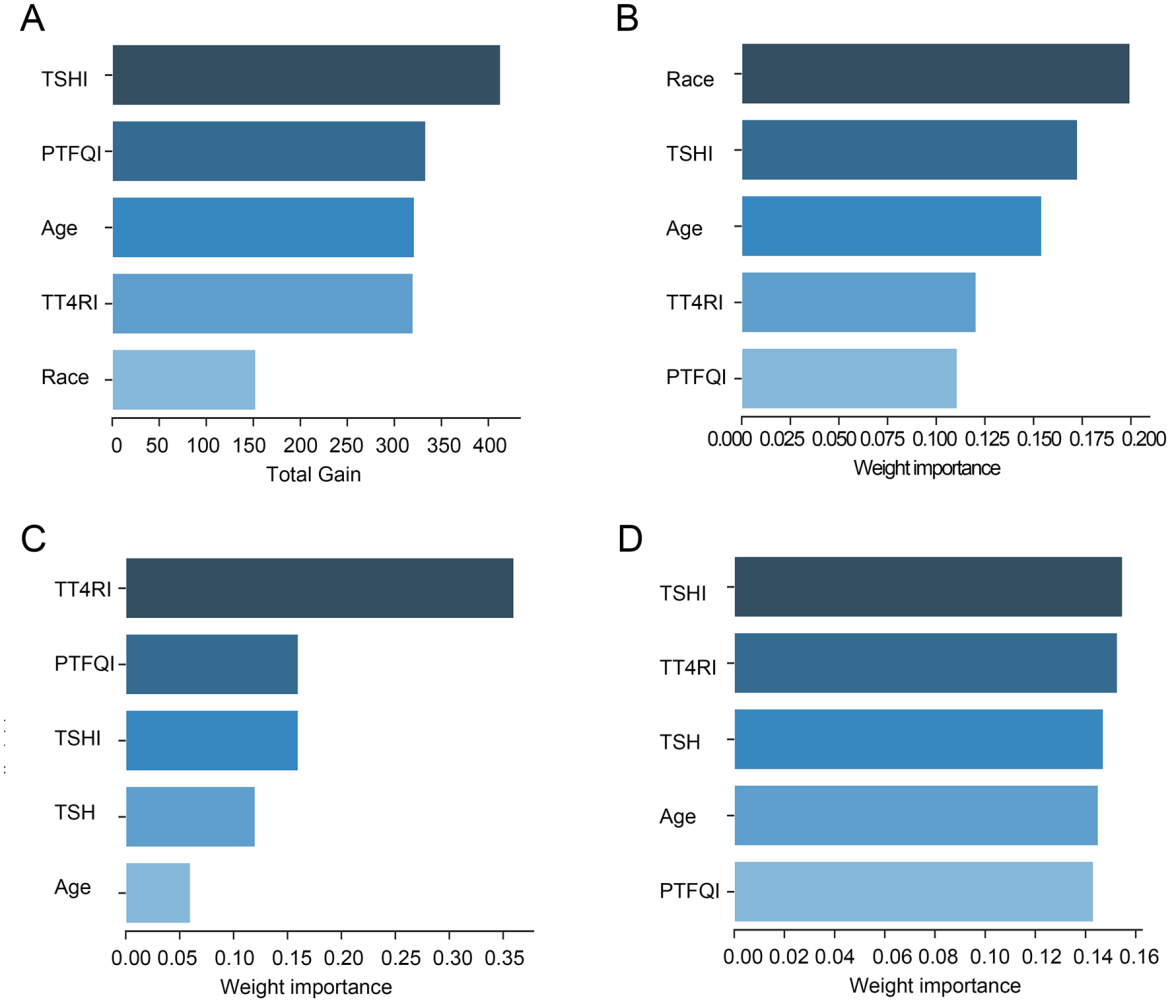
The importance ranking bar chart of the top five factors via four methods. (**A**) Xgboost (**B**) AdaBoost (**C**) Random forest (**D**) light GBM.

The above results have demonstrated the significant association between 4 indices and sleep duration. Finally, we deeply explored their importance on the depression score via different methods including Xgboost, AdaBoost, Random Forest, and light Gradient Boosting Machine (GBM). We found that the importance of TSHI ranked first in Xgboost and light GBM (Fig. 4A,D). TSHI was second only to age in AdaBoost (Fig. 4B). In the Random forest, TSHI ranked the three (Fig. 4C). Taken together, the importance degree of TSHI was relatively higher among 4 indices associated with sensitivity to thyroid hormones.

## Discussion

In patients with depression, severe sleep problems are often manifested. Southmayd et al. indicated that major depression patients were able to alleviate sleep deprivation by receiving thyroxine^27^. The study revealed the influence of thyroid hormones on sleep in patients with severe depression. However, those studies were limited to the relationship between sleep duration and thyroid function, and did not reveal the association of thyroid-related hormones with sleep duration in low-grade depression populations. Currently, there is limited research revealing the correlation between thyroid-related hormones and sleep duration in this specific group, which warrants further investigation.

In this study, we aimed to explore the association between thyroid-related hormone indexes and sleep duration in populations with depression. We found that there was a positive correlation between thyroid-related hormone indexes and sleep duration just in populations with depression degrees lower than Moderate. No significant association was observed in the subgroup with a greater than moderate degree. It followed that the findings from these studies may not be applicable for people experiencing depression with different degrees. More importantly, we found that only indicators reflecting the sensitivity to thyroid hormones (PTFQI, TSH, TT4RI, and TSHI) but not common T3 and T4, were significantly related to the sleep duration in this subgroup populations, suggesting that thyroid hormone sensitivity indexes may be more suitable for evaluating the sleep duration in depression, compared with these common thyroid-related hormone indexes. These results further highlighted the importance of sensitivity to thyroid hormones. To our knowledge, this is the first study to explore the association between the sensitivity to thyroid hormones and sleep duration in particular population.

Further, we sufficiently justified the association between these 4 indicators and sleep duration in the subgroup populations. With the increase of these 4 indicators, the sleep duration increased. These results suggested the negative association between longer sleep duration and poor sensitivity to thyroid hormones. Especially, TSHI was relatively more important among these indicators, which reflect the sensitivity of FT4 to pituitary^16,28^. These results highlighted the importance of thyroid hormone homeostasis on the sleep.

The importance of thyroid-related hormone on the sleep has been widely reported. Wang et al. indicated that there was a negative relationship between FT3 levels and sleep duration^14^. The T3, T4, and TSH levels were positively correlated with the severity of insomnia symptoms^29^. Song et al.^30^ have shown that decreased thyroid hormone levels can lead to difficulty in falling asleep and reduced sleep duration. Tian et al. showed that increased T4 levels can stimulate symptoms similar to restless legs syndrome, leading to insomnia and reduced sleep duration^31^. The potential mechanism of thyroid hormone involved in the sleep needs to be revealed. It is well known that thyroid function is closely related to depression, and sleep disorder is a common complication in patients with depression. The hypothalamic–pituitary–adrenal (HPA) and hypothalamic-pituitary-thyroid (HPT) axis changes in patients with depression, both of which are closely related to sleep. The HPT axis is involved in the occurrence and development of sleep. For example, patients with thyroid dysfunction usually will have insomnia, hypersomnia, etc. Intravenous injection of thyrotropin-releasing hormone (TRH) can change sleep parameters^28,32^. The activity of the HPA axis is related to the excessive awakening of insomnia^33,34^. Studies have shown that in patients with depression, the hippocampus, the negative feedback center of the HPA axis, was impaired in the negative feedback regulation of glucocorticoids through the HPA axis, resulting in increased cortisol. Then, glucocorticoids activated TRH neurons, resulting in increased TRH secretion, thereby down-regulating TRH receptors on thyrotrophs. Therefore, in depressive patients with normal thyroid function, thyroid function has changed, including increased T4 levels, decreased TSH response to TRH, and no increase in nocturnal TSH^35^.

Conversely, sleep duration can also affect the levels of thyroid hormones. Kim et al. confirmed that long sleep duration and short sleep duration were risk factors for increased and decreased TSH, respectively, compared to optimal sleep duration, based on data from KNHANES in Korea^36^. The reason is that the rhythm of TSH secretion may be related to melatonin which is the main hormone secreted by pineal stromal cells. There is a large number of melatonin receptors expressed in the anterior pituitary, and the change in light duration will affect the effect of melatonin on the anterior pituitary.

The study had some limitations. First, it is possible that the result was not an exact causal relationship due to cross-sectional analysis. Continuity research should be carried out in the future.

Second, we did not explore the level of adrenaline and other hormones. Third, the effect of drugs on thyroid hormones was not ruled out due to the lack of medication information.

## Conclusion

To sum up, sensitivity to thyroid hormones was significantly related to the sleep duration in the euthyroid populations with depression degree lower than moderate. Among the subgroup populations, the poor sensitivity to thyroid hormones correlated with longer sleep duration. This study highlighted the importance of thyroid hormone homeostasis on the sleep in depression (Supplementary Information S1).

### Supplementary Information

Supplementary Information.

## Data Availability

All data generated or analysed during this study are included in this supplementary information files.
